# Predicting Delayed Neurocognitive Recovery After Non-cardiac Surgery Using Resting-State Brain Network Patterns Combined With Machine Learning

**DOI:** 10.3389/fnagi.2021.715517

**Published:** 2021-11-12

**Authors:** Zhaoshun Jiang, Yuxi Cai, Xixue Zhang, Yating Lv, Mengting Zhang, Shihong Li, Guangwu Lin, Zhijun Bao, Songbin Liu, Weidong Gu

**Affiliations:** ^1^Department of Anesthesiology, Huadong Hospital Affiliated to Fudan University, Shanghai, China; ^2^Shanghai Key Laboratory of Clinical Geriatric Medicine, Shanghai, China; ^3^Center for Cognition and Brain Disorders, The Affiliated Hospital of Hangzhou Normal University, Hangzhou, China; ^4^Department of Radiology, Huadong Hospital Affiliated to Fudan University, Shanghai, China; ^5^Department of Geriatric Medicine, Huadong Hospital Affiliated to Fudan University, Shanghai, China; ^6^Research Center on Aging and Medicine, Fudan University, Shanghai, China

**Keywords:** delayed neurocognitive recovery, default mode network, functional connectivity, machine learning, resting-state functional MRI

## Abstract

Delayed neurocognitive recovery (DNR) is a common subtype of postoperative neurocognitive disorders. An objective approach for identifying subjects at high risk of DNR is yet lacking. The present study aimed to predict DNR using the machine learning method based on multiple cognitive-related brain network features. A total of 74 elderly patients (≥ 60-years-old) undergoing non-cardiac surgery were subjected to resting-state functional magnetic resonance imaging (rs-fMRI) before the surgery. Seed-based whole-brain functional connectivity (FC) was analyzed with 18 regions of interest (ROIs) located in the default mode network (DMN), limbic network, salience network (SN), and central executive network (CEN). Multiple machine learning models (support vector machine, decision tree, and random forest) were constructed to recognize the DNR based on FC network features. The experiment has three parts, including performance comparison, feature screening, and parameter adjustment. Then, the model with the best predictive efficacy for DNR was identified. Finally, independent testing was conducted to validate the established predictive model. Compared to the non-DNR group, the DNR group exhibited aberrant whole-brain FC in seven ROIs, including the right posterior cingulate cortex, right medial prefrontal cortex, and left lateral parietal cortex in the DMN, the right insula in the SN, the left anterior prefrontal cortex in the CEN, and the left ventral hippocampus and left amygdala in the limbic network. The machine learning experimental results identified a random forest model combined with FC features of DMN and CEN as the best prediction model. The area under the curve was 0.958 (accuracy = 0.935, precision = 0.899, recall = 0.900, F1 = 0.890) on the test set. Thus, the current study indicated that the random forest machine learning model based on rs-FC features of DMN and CEN predicts the DNR following non-cardiac surgery, which could be beneficial to the early prevention of DNR.

**Clinical Trial Registration:** The study was registered at the Chinese Clinical Trial Registry (Identification number: ChiCTR-DCD-15006096).

## Introduction

Delayed neurocognitive recovery (DNR) is one of the most frequent neurological complications in elderly patients after major non-cardiac surgery (Evered et al., 2018; Zhang et al., 2018). Delayed neurocognitive recovery is defined as a decline in cognitive performance within the first month after surgery. It has been demonstrated that DNR increases the incidence of postoperative complications and prolongs recovery time (Chen and Qin, 2020). The International study of postoperative cognitive dysfunction (ISPOCD1) reported a 25.8% incidence of cognitive dysfunction at 1 week and 9.9% at 3 months in patients aged over 60-years-old after non-cardiac surgery (Moller et al., 1998). Considering the high incidence of and the poor outcomes associated with DNR, it is essential to identify the potential predictors of DNR and construct an objective prediction model to screen patients at a high risk of DNR.

Recently, resting-state functional magnetic resonance imaging (rs-fMRI) has been employed to explore the potential imaging biomarkers in various cognitive disorders (Xue C. et al., 2019). Resting-state functional magnetic resonance imaging detects regional spontaneous brain activity and the functional integrity of brain networks without any task or stimulus non-invasively. The amplitude of low-frequency fluctuation reflected localized neural activity and was analyzed in our previous study to identify the neuroimaging risk factors for DNR. The previous analyses found that elderly patients with altered preoperative regional neural activity in the bilateral middle cingulate cortex (MCC) were more susceptible to DNR following non-cardiac surgery (Jiang et al., 2020). The estimation of the regional pairwise correlation of blood oxygen level-dependent signals facilitated rs-fMRI to characterize the intrinsic brain network architecture (Smith et al., 2013). Functional connectivity (FC) analysis was performed to explore the underlying associations between cognitive impairment and connectivity patterns of specific brain networks, such as the default mode network (DMN), limbic network, salience network (SN), and central executive network (CEN). For example, a study using rs-fMRI reported that the functional integrity of the DMN and CEN was affected in the Alzheimer’s disease (AD) group compared to the controls (Ripp et al., 2020). Therefore, the present study is a reanalysis of previously published data. We also further investigated whether subjects with postoperative DNR had abnormal connectivity in the multiple cognitive-related brain networks before surgery using seed-based whole-brain FC analysis.

With rapid progress in artificial intelligence, machine learning, a data-driven pattern of computer-aided recognition techniques, is increasingly applied for medical diagnosis (Zhu et al., 2020). Moreover, machine learning could incorporate diversified neuroimaging features and identify critical factors or interactions that were previously unknown; this might improve the accuracy of the model (Solomon et al., 2020). Due to the advantages, such as universality and accuracy, a large number of studies assessed the application of machine learning to rs-fMRI data for clinical diagnosis and prediction of neuropsychological disorders, such as AD and postoperative delirium (Asgari et al., 2020; Wang et al., 2020; Zhu et al., 2021). However, no model has been established for the prediction of DNR using machine learning combined with neuroimaging data.

The present study aimed to explore the preoperative differences in seed-based FC in the multiple cognitive-related brain networks between patients with and without DNR and develop a machine learning model based on neuroimaging data to predict the DNR after non-cardiac surgery.

## Materials and Methods

### Ethics Approval and Participants

This study was approved by the Ethics Committee of Huadong Hospital Affiliated to Fudan University, Shanghai, China (approval number: 20170020), and written informed consent was obtained from all subjects participating in the trial registered on the Chinese Clinical Trial Registry (^1^ Identification number: ChiCTR-DCD-15006096, Principal Investigator: Weidong Gu, Date of registration: March 16, 2015).

The inclusion and exclusion criteria of our study population have been described previously (Jiang et al., 2020). The inclusion criteria included patients scheduled to undergo non-cardiac surgery, age ≥ 60-years-old, American Society of Anesthesiologists (ASA) classification I-III, and right handedness. The exclusion criteria included baseline mini-mental state examination (MMSE) score <24 points, education duration <6 years, preexisting mental or psychiatric disease, cardiac or central nervous system vascular disease, Parkinson’s disease, cardiac or cranial surgery history, major surgery in past 12 months, taking sedatives or antidepressants during the nearest year, alcohol or drug abuse, vision and audition impairment or language troubles impeding communication, and situations unsuitable for MRI examination. This manuscript adheres to the applicable strengthening the reporting of observational studies in epidemiology (STROBE) guidelines.

### Neurocognitive Assessment

We used the Z score method recommended by the ISPOCD1 to diagnose DNR for each patient (Moller et al., 1998). This is a standardized method for the identification of postoperative neurocognitive disorders that have been described in a previous study (Jiang et al., 2020). All patients underwent comprehensive neurocognitive tests 1 day before surgery (baseline) and at 7-14 days after the surgery (follow-up). The comprehensive tests included MMSE, verbal fluency test (VFT), digit symbol substitution test (DSST), digit span forward and backward test (DSF/DSB), and trail making test-part A (TMT-A). For each test, we compared the changes (Δx) from baseline scores to follow-up scores in patients. To correct the learning effect, we recruited 30 volunteers, age- and education duration- matched to the surgical patients, who completed the same neurocognitive tests as those performed on the surgical patients. For volunteers, we calculated the changes from baseline scores to follow-up scores to obtain the average learning effects (Δxc) and the standard deviations [SD(Δxc)] in each test. The Z score was built as follows: (Δx-Δxc)/SD(Δxc). To create a composite Z score, the Z scores of all six tests in an individual patient were summarized and divided by the standard deviations for the sum of Z scores in volunteers. The composite Z score was built as follows: ΣZ/SD(ΣZc). A patient was diagnosed as having DNR when the Z scores of at least two of the neurocognitive tests or the composite Z score were ≥1.96.

### Resting-State Functional Magnetic Resonance Imaging Data Acquisition

All MRI data were acquired on a Siemens Skyra 3.0 T MRI scanner before surgery. The complete MRI acquisition protocol included three-dimensional (3D) anatomical T1-weighted imaging and fMRI echo-planar imaging. The 3D anatomical T1-weighted imaging parameters were as follows: 176 sagittal slices, repetition time = 1900 ms, echo time = 3.57 ms, voxel size = 1 × 1 × 1 mm, and flip angle = 9°. The echo-planar imaging sequence parameters were as follows: 33 axial slices, slices thickness = 4 mm with a 0-mm gap, repetition time = 3000 ms, echo time = 30 ms, voxel size = 3.4 × 3.4 × 4 mm, and flip angle = 90°. During the fMRI imaging, 120 volumes were obtained that lasted 8.5 min.

### Image Preprocessing and Seed-Based Functional Connectivity Analysis

All rs-fMRI data were processed using Statistical Parametric Mapping version 12, RESTplus version 1.22, and BrainNet viewer, based on MATLAB version R2013b (Ren et al., 2016). We discarded the first five volumes to avoid the potential noise related to the participants’ adaptation to the scanner. The remaining images preprocessing included slice timing, head motion correction, spatial normalization, smoothing (6mm full-width half-maximum Gaussian kernel), low-frequency filtering (0.01-0.08 Hz), linear trend removal, and nuisance covariates regression (motion artifact, white matter signal, and cerebrospinal fluid signal) (Jiang et al., 2020).

A seed-based analysis was performed to explore the preoperative whole-brain voxel-wise FC alteration in the main nodes from multiple cognitive-related networks in DNR patients. To identify the regions of interest (ROIs) in the present study, eighteen spherical ROIs with a 6-mm radius from the DMN, limbic network, SN, and CEN, were adopted based on previous studies (Supekar et al., 2009; Fan et al., 2018; Liu et al., 2020) (see the detailed ROI Montreal Neurological Institute coordinates in Supplementary Table 1). For the FC analysis, we extracted the mean time series of each ROI and correlated it with the time series of each voxel of the whole brain. Then, Fisher’s r-to-z transformation was used to improve the normality of the correlation coefficients.

The group comparisons of rs-fMRI data were performed using two-sample *t*-tests, with age, sex, and duration of education as covariates. The data were corrected using cluster-based false discovery rate (FDR) with uncorrected voxel *P* < 0.001 and corrected cluster *P* < 0.05 (Chumbley and Friston, 2009).

### Statistical Analysis and Machine Learning

Analyses of patient characteristics and neurocognitive data were performed using SPSS 22.0 and GraphPad Prism. According to the distribution of the data, two-sample *t*-test or Mann-Whitney test was used to assess the differences in continuous variables between the DNR and non-DNR groups. Categorical variables were analyzed using the chi-square test. The differences in neurocognitive test scores at days 7-14 follow-up between the patients with and without DNR were compared, with baseline scores as a covariate. A *P*-value < 0.05 was adopted as the criterion to indicate statistical significance.

Based on the rs-fMRI variables, predictive models of DNR were established using the support vector machine algorithm, decision tree algorithm, and random forest (RF) algorithm. The dataset was randomly divided into a training set containing 70% of the samples and a test set containing the remaining 30%. Next, the effect of each model was assessed by 10-fold cross-validation on the training set, which could reduce selection bias or overfitting. The comparison of their classification performance identified the model with the best predictive efficacy. Subsequently, due to the high dimensionality of the rs-fMRI data, we evaluated the corresponding weights of the indicators in the model to remove irrelevant or unimportant information from the data, thereby improving the generalization ability of the DNR prediction model. In addition, because of the minority of DNR patients in the data set, a separate experiment was conducted using the Synthetic Minority Oversampling Technique (SMOTE) to enrich the data. This is an advanced oversampling method that generates synthetic samples in the minority class of imbalanced datasets to avoid overfitting (Decaro et al., 2021; Popoola et al., 2021).

Furthermore, we selected the model with the best classification performance and the strongest generalization ability and adjusted the parameters to obtain that with the best predictive efficacy. Finally, the predictive performance was evaluated in the test set based on the best model with optimal parameters. The classification performance of the machine learning algorithms was evaluated using the statistical metrics of precision, recall, F1, accuracy, and area under the receiver operating characteristic curve (AUC-ROC). F1 is a weighted average of precision and recall (Wang et al., 2020), which can comprehensively evaluate the balance of model performance between precision and recall. A higher F1 value indicates a satisfactory performance of the model. The statistical analyses of machine learning algorithms were performed in Python.

## Results

### Subject Characteristics and Neurocognitive Results

In this study, 74 patients completed both the rs-fMRI scan and the neurocognitive assessment follow-up. The flow-chart of patient selection in this study is provided in Supplementary Figure 1. A total of 16 patients were diagnosed with DNR, and the incidence of DNR was 21.6% at 7-14 days post-surgery. The characteristics and neurocognitive results of the subjects are summarized in Table 1. We found that the non-DNR subjects showed a significantly longer education duration than the DNR subjects (*P* = 0.002). However, no differences were detected in the other baseline and intraoperative data between the two groups.

**TABLE 1:** Characteristics and neurocognitive results.

	All subjects	DNR	non-DNR	*P* value
Number of subjects	74	16	58	-
Age (years)	64.0 (61.8, 68.0)	63.5 (62.0, 67.0)	64.0 (61.0, 68.3)	0.598^b^
Sex (male/female)	41/33	12/4	29/29	0.075^c^
Education (years)	9 (9, 12)	6 (6, 9)	9 (9, 12)	0.002^b^
Height (m)	1.68 (1.60, 1.72)	1.70 (1.63, 1.73)	1.65 (1.59, 1.71)	0.185^a^
Weight (kg)	60.0 (53.8, 70.0)	59.0 (50.0, 70.5)	60.0 (54.8, 70.0)	0.324^b^
BMI ≥ 24 (n,%)	22 (29.7)	3 (18.8)	19 (32.8)	0.437^c^
Smoking (n,%)	22 (29.7)	7 (43.8)	15 (25.9)	0.281^c^
Surgical history (n,%)	34 (45.9)	6 (37.5)	28 (48.3)	0.444^c^
**Comorbidities (n,%)**				
Hypertension	31 (41.9)	7 (43.8)	24 (41.4)	0.865^c^
Diabetes mellitus	8 (10.8)	1 (6.3)	7 (12.1)	0.835^c^
Anemia	15 (20.3)	5 (31.3)	10 (17.2)	0.309^c^
COPD	19 (25.7)	5 (31.3)	14 (24.1)	0.800^c^
Peptic ulcer disease	9 (12.2)	4 (25.0)	5 (8.6)	0.179^c^
**Intraoperative conditions**				
Surgical duration (min)	106 (72, 149)	120 (114, 166)	95 (70, 135)	0.055^b^
Minimally invasive/open surgery	66/8	12/4	54/4	0.107^c^
Propofol (mg)	324 (60, 700)	300 (53, 844)	324 (68, 678)	0.974^b^
Sufentanil (μg)	30 (14, 35)	35 (21, 49)	30 (25, 35)	0.148^b^
Remifentanil (mg)	1.3 (0.8, 2.0)	1.5 (1.3, 2.1)	1.2 (0.7, 1.8)	0.059^b^
**Neurocognitive followup**				
MMSE	27.5 (26.0, 28.0)	26.0 (23.3, 27.8)	28.0 (26.0, 28.3)	0.032^a^
VFT	14.5 (13.0, 19.0)	12.5 (10.0, 14.0)	15.0 (13.8, 19.0)	0.002^a^
DSST	28.0 (21.0, 35.0)	19.5 (15.0, 24.0)	30.0 (24.8, 35.0)	<0.001^a^
DSF	8.0 (7.0, 8.0)	6.5 (5.0, 8.0)	8.0 (7.0, 8.0)	0.008^a^
DSB	4.0 (3.0, 4.0)	3.0 (3.0, 4.0)	4.0 (3.0, 4.0)	<0.001^a^
TMT-A	44.5 (35.8, 61.0)	76.0 (48.5, 104.5)	42.0 (33.0, 54.0)	<0.001^a^

**P-*value refers to group comparison of DNR group vs. non-DNR group by ^a^ *t*-test; ^b^ Mann-Whitney U test; ^c^ chi-square test.*

*BMI, body mass index; COPD, chronic obstructive pulmonary disease; DNR, delayed neurocognitive recovery; DSB, digit span backward; DSF, digit span forward; DSST, digit symbol substitution test; MMSE, mini-mental state examination; TMT-A, trail making test-part A; VFT, verbal fluency test.*

As expected, after adjusting for the baseline neurocognitive test scores, we observed that the DNR patients exhibited significantly lower MMSE, VFT, DSST, DSF, and DSB follow-up scores and higher TMT-A follow-up scores (all *P* < 0.05) compared to the non-DNR patients. These results indicated that the DNR group has a poor performance after non-cardiac surgery in many cognitive function domains, such as working memory, attention, executive function, and visual-spatial ability.

### Altered Seed-Based Functional Connectivity Patterns of Multiple Cognitive-Related Networks

**FIGURE 1:**
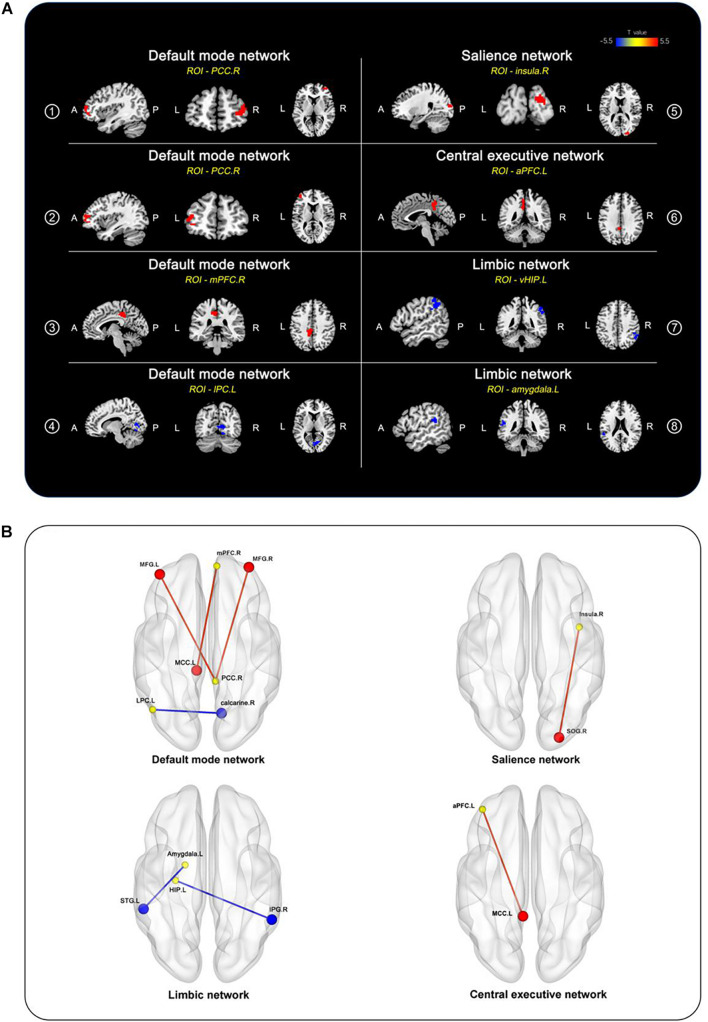
Brain regions of between-group differences in FC after adjusting for age, sex, and education duration (cluster *P* < 0.05, FDR-corrected). **(A)** Compared to the non-DNR group, the DNR group exhibited aberrant connectivity to the whole brain in seven ROIs, including the right PCC, right mPFC, and left lateral parietal cortex located in the DMN; the right insula located in the SN; the left anterior prefrontal cortex in the CEN; the left ventral hippocampus and left amygdala in the limbic network. The color scale denotes the T values. **(B)** 3D view of the brain. The yellow nodes represent the ROIs in the DMN, SN, CEN, and limbic network. The red/blue nodes and edges represent the brain regions with increased/decreased FC to the ROIs in the DNR patients compared to the non-DNR patients. aPFC, anterior prefrontal cortex; CEN, central executive network; DMN, default mode network; DNR, delayed neurocognitive recovery; FC, functional connectivity; FDR, false discovery rate; vHIP, ventral hippocampus; IPG, inferior parietal gyrus; LPC, lateral parietal cortex; MCC, middle cingulate cortex; MFG, middle frontal gyrus; mPFC, medial prefrontal cortex; PCC, posterior cingulate cortex; ROIs, regions of interest; SN, salience network; SOG, superior occipital gyrus; STG, superior temporal gyrus; A, anterior; P, posterior; L, left; R, right.

After adjusting for age, sex, and education duration, significant differences were detected in the preoperative whole-brain connectivity of seven ROIs from the DMN, SN, CEN, and limbic network between the two groups (Figure 1 and Table 2). In the DMN, the DNR group exhibited higher FC ➀ between the right posterior cingulate cortex (PCC) and right middle frontal gyrus, ➁ between the right PCC and left middle frontal gyrus, ➂ between the right medial prefrontal cortex (mPFC) and left MCC, and ➃ lower FC between the left lateral parietal cortex and right calcarine compared to the non-DNR group. Also, the DNR patients had higher FC ➄ between the right insula in the SN and right superior occipital gyrus, and ➅ between the left anterior prefrontal cortex in the CEN and left MCC. Additionally, regarding the altered patterns of the limbic network, decreased connections were detected in the DNR group compared to the non-DNR group ➆ between the left ventral hippocampus and right inferior parietal gyrus, and ➇ between the left amygdala and left superior temporal gyrus. The data were corrected using the cluster-based FDR (uncorrected voxel *P* < 0.001 and corrected cluster *P* < 0.05).

**TABLE 2:** Brain regions showing seed-based FC differences before surgery between the DNR and non-DNR groups.

	ROIs	Brain regions	Direction	MNI coordinates (x/y/z mm)	Cluster size	PeakT
	**Default mode network (DMN)**					
➀	Posterior cingulate cortex.R	middle frontal gyrus.R	DNR > Non-DNR	36 54 6	48	3.968
➁	Posterior cingulate cortex.R	middle frontal gyrus.L	DNR > Non-DNR	–42 48 12	48	4.604
➂	Medial prefrontal cortex.R	middle cingulate cortex.L	DNR > Non-DNR	–9 –33 42	88	5.106
➃	lateral parietal cortex.L	calcarine.R	DNR < non-DNR	12 –69 6	105	–5.252
	**Salience network (SN)**					
➄	Insula.R	superior occipital gyrus.R	DNR > Non-DNR	24 –90 15	37	4.747
	**Central executive network (CEN)**					
➅	Anterior prefrontal cortex.L	middle cingulate cortex.L	DNR > Non-DNR	–6 –45 36	66	4.659
	**Limbic network**					
➆	Ventral hippocampus.L	inferior parietal gyrus.R	DNR < non-DNR	54 -48 42	111	–4.750
➇	Amygdala.L	superior temporal gyrus.L	DNR < non-DNR	–54 –39 21	37	–4.506

*CEN, central executive network; DMN, default mode network; DNR, delayed neurocognitive recovery; FC, functional connectivity; MNI, Montreal Neurological Institute; ROIs, regions of interest; SN, salience network; R, right; L, left.*

### Machine Learning Prediction Models

Using a randomization method, 70% of the total samples were divided into the training set and 30% into the test set. The optimal model was established through 10-fold cross-validation on the training set, and each predictive model was evaluated with accuracy, precision, recall, and F1 metrics. To establish the DNR prediction models, the three machine learning algorithms included eight rs-fMRI variables (➀-➇; Table 2) and subject characteristics (age, sex, and education). The following metrics with default parameters were identified in the training set: support vector machine algorithm (precision = 0.804, recall = 0.782, F1 = 0.779, accuracy = 0.905); decision tree algorithm (precision = 0.698, recall = 0.707, F1 = 0.687, accuracy = 0.837); RF algorithm (precision = 0.849, recall = 0.811, F1 = 0.813, accuracy = 0.907). The results indicated that the RF algorithm using rs-fMRI data combined with age, sex, and education achieved the best performance for predicting DNR following non-cardiac surgery.

**FIGURE 2:**
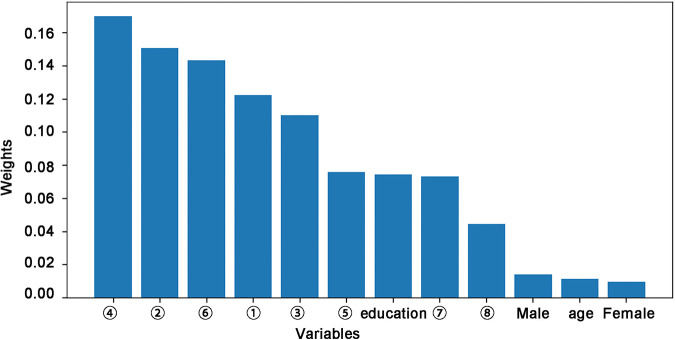
Feature importance across rs-fMRI features and demographic data included in the random forest algorithm for the prediction of DNR. ➀-➇ represents a number of FC variables: ➀ PCC.R–middle frontal gyrus.R; ➁ PCC.R–middle frontal gyrus.L; ➂mPFC.R–MCC.L; ➃ Lateral parietal cortex.L–calcarine.R; ➄ Insula.R–superior occipital gyrus.R; ➅Anterior prefrontal cortex.L–MCC.L; ➆ Ventral hippocampus.L–inferior parietal gyrus.R; ➇ Amygdala.L–superior temporal gyrus.L. DNR, delayed neurocognitive recovery; FC, functional connectivity; MCC, middle cingulate cortex; mPFC, medial prefrontal cortex; PCC, posterior cingulate cortex; rs-fMRI, resting-state functional MRI; R, right; L, left.

The RF algorithm also provides the corresponding weight of each variable, thereby identifying the variable that influences the predictions (Corradi et al., 2018). Figure 2 shows the corresponding weights of each variable calculated by the RF algorithm to reveal the contributions of these variables to the model. The crucial factors were ➃FC of the left lateral parietal cortex – right calcarine, ➁FC of the right PCC – left middle frontal gyrus, ➅FC of the left anterior prefrontal cortex – left MCC, ➀FC of the right PCC – right middle frontal gyrus, and ➂FC of the right mPFC – left MCC. The contribution of these five variables to the DNR model was close to 70%, indicating that the whole-brain FC of the DMN and CEN were critical influencing factors for the development of DNR.

Next, the machine learning modeling was continued to predict the DNR according to the data of feature screening. The rs-fMRI data related to DMN and CEN were extracted, combined with the RF algorithm and SMOTE method to construct the DNR prediction model and compare the prediction performance of (1) initial model (all variables); (2) initial model + SMOTE; (3) simplified model (only FC data of DMN and CEN); (4) simplified model + SMOTE. The following metrics were identified as follows: (1) initial model (precision = 0.849, recall = 0.811, F1 = 0.813, accuracy = 0.907); (2) initial model + SMOTE (precision = 0.874, recall = 0.851, F1 = 0.848, accuracy = 0.921); (3) simplified model (precision = 0.836, recall = 0.802, F1 = 0.799, accuracy = 0.882); (4) simplified model + SMOTE (precision = 0.852, recall = 0.830, F1 = 0.823, accuracy = 0.890). The results showed that the prediction performance changed slightly when the feature number decreased significantly to 5. This phenomenon indicated that the simplified RF model could achieve good prediction performance for DNR. In addition, SMOTE oversampling method improved the prediction efficacy.

**FIGURE 3:**
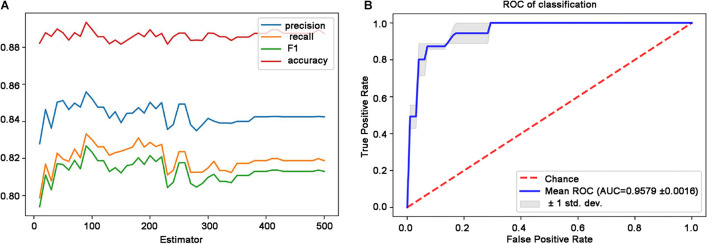
Performance of the simplified RF model. **(A)** Classification performance with respect to the distinct number of decision trees. **(B)** ROC curves and the AUC of the RF model are based on the 90 decision trees. Blue line denotes the average ROC curve, and the shaded area indicates the lower and upper limits of the AUC in the 10-fold cross-validation. AUC, area under the ROC curve; RF, random forest; ROC, receiver operating characteristic.

Based on DMN and CEN data using SMOTE method, this study identified the optimal parameters by adjusting the number of decision trees in the simplified RF model. Figure 3A shows that the best classification accuracy was obtained when the number of decision trees was 90. Finally, the RF classifier accuracy was determined as high as 0.935 (precision = 0.889, recall = 0.900, F1 = 0.890) in the test set. Based on the 90 decision trees to build the model, a high forecast performance of the model was obtained with an average AUC of 0.958 in 10-fold cross-validation, indicating an adequate average effect of the model. The lower and upper limits of the AUC in the cross-validation were 0.956 and 0.960, respectively, which indicates a stable model (Figure 3B).

In summary, the simplified RF model based on the FC data of DMN and CEN could effectively predict the DNR following the non-cardiac surgery.

## Discussion

To the best of our knowledge, this is the first study using machine learning combined with the neuroimaging data to predict the DNR following non-cardiac surgery. In the present study, we reported two significant findings. First, the DNR patients exhibited altered preoperative whole-brain functional connectivity patterns of the DMN, limbic network, SN, and CEN. Second, the RF machine learning model showed that multiple cognitive-related network fMRI data could be used to predict postoperative DNR with high accuracy.

Due to the low sensitivity and specificity of biological markers, brain function research based on rs-fMRI is a breakthrough point in the study of cognitive dysfunction diseases. Several studies have applied rs-fMRI as one of the main methods to illuminate the mechanisms underlying cognitive disorders. The most studied connectivity patterns of some cognitive-related networks are the DMN, SN, CEN, and limbic network. DMN is related to cognition processing, and the core hubs are the mPFC and the PCC. Fan et al. performed a seed-based analysis with seeds located in the classical DMN regions, including bilateral mPFC and PCC, and found that the DMN-involved FC alterations were correlated with sustained attention deficits (Fan et al., 2018). The lateral parietal cortex is also a key area of DMN. In a seed-based FC study to explore the association between DMN and schizophrenia with cognitive impairment, Dauvermann et al. selected mPFC, PCC, and lateral parietal lobes as the seeds of the DMN and reported patients with cognitive impairment had reductions in FC between the left/right parietal lobe and multiple other regions (Dauvermann et al., 2021). The SN anchored in the insula and anterior cingulate cortex plays a crucial role in identifying the cognitively relevant events that guide attention, detection, emotional information, and orientation (Menon and Uddin, 2010). The CEN participates in high-level cognitive functions, including decision-making, information processing, executive function, and working memory. Its core nodes include the anterior prefrontal cortex and superior parietal lobule (Viard et al., 2019; Xue J. et al., 2019). We also analyzed the connection pattern between the limbic network (hippocampus and amygdala) and each voxel of the whole brain in DNR patients. Therefore, we used seed-based methods to explore whole-brain voxel-wise FC alterations in DNR by choosing the main nodes as ROIs in these networks and integrated the identified abnormal FC changes by machine learning methods to establish a model to predict DNR in elderly patients.

In recent years, the combination of machine learning and medical events has become a hot topic. It has achieved productive results, given that the machine learning algorithms can accommodate all variables simultaneously and model their interactions to optimize the between-group classification (Oh et al., 2018). Also, the application of machine learning has also been reported for the prediction of postoperative complications (Barber et al., 2021; Bolourani et al., 2021). A previous study demonstrated that applying complex or simple machine learning algorithms improves the prediction of postoperative delirium after cardiac surgery, which reduces the costs by preventing postoperative complications and optimizes patient outcomes (Mufti et al., 2019). In this study, we built an RF model based on FC features of multiple cognitive-related networks to predict the postoperative DNR and achieved acceptable accuracy, precision, recall, and F1.

In the present study, the RF model identified critical factors for the occurrence of DNR: CEN and DMN. The anterior prefrontal cortex is the key region of the CEN. The results showed that DNR patients had higher preoperative connectivity between the anterior prefrontal cortex and MCC, and the contribution of the feature to the DNR model was about 15%. In addition, we also found a high preoperative FC between mPFC, which is the core region of the DMN, and MCC in the DNR patients. These results indicated that MCC has abnormal functional connections with key seed points in CEN and DMN. The MCC is a crucial part of the limbic network, involved in various cognitive functions, such as attention, working memory, and executive function (Shackman et al., 2011; Yuan et al., 2016; Zheng et al., 2018). Reportedly, MCC was activated while performing working memory tasks and during divided attention (Petit et al., 1998; Bush et al., 2005). In addition to working memory and attention function, MCC is also involved in executive function. Yuan et al. demonstrated that MCC is a core region of the executive function network that mediates episodic memory processing (Yuan et al., 2016). Several clinical studies have reported that patients with abnormal regional neural activity and functional connections of the MCC were susceptible to cognitive impairment. Li et al. demonstrated that patients with mild cognitive impairment have higher spontaneous synchrony in the MCC compared to healthy subjects (Li et al., 2020). In an fMRI study evaluating the FC patterns throughout the progression of AD, Skouras et al. observed a stronger FC between the MCC and several brain regions in the preclinical asymptomatic and mild cognitive impairment group (Skouras et al., 2019). In a previous analysis, we deduced that altered neural activity and whole-brain connectivity pattern of MCC were independent risk factors for DNR following non-cardiac surgery (Jiang et al., 2020). Taken together, these findings indicated that the local neural activity of MCC and the FC pattern between MCC and other brain networks (DMN and CEN) plays crucial roles in the pathogenesis of various diseases related to cognitive dysfunction.

The abnormal FC patterns of DMN were associated with various diseases related to cognitive impairment, such as mild cognitive impairment, AD, Parkinson’s disease, and attention-deficit/hyperactivity disorder (Zhang H. et al., 2020; Duffy et al., 2021; Luo et al., 2021; Tang et al., 2021; Wang et al., 2021). Zhang et al. investigated the subjects at high risk of AD compared to the low-risk subjects, presenting significantly altered FC in the PCC and middle frontal cortex (Zhang X. Y. et al., 2020). We also found that DNR patients showed a higher preoperative FC between PCC and bilateral middle frontal cortex compared to the non-DNR patients. Notably, the critical factor for DNR occurrence was the FC of the lateral parietal cortex–calcarine. The calcarine is a part of the primary visual cortex contributing to visual-spatial processing, attention, episodic memory, and information maintenance about a stimulus in working memory (Pratte and Tong, 2014; Bergmann et al., 2016; Cho et al., 2018). The abnormal alterations in FC of calcarine were related to cognitive impairment (Moon and Jeong, 2017). In a previous study, we also found that altered preoperative FC of calcarine was independently associated with the occurrence of DNR (Jiang et al., 2020). The DSST and TMT-A neurocognitive tests were employed to assess the visual-related cognitive function (Joy et al., 2003; Llinàs-Reglà et al., 2017). Herein, we found that the DNR patients showed lower DSST and higher TMT-A scores postoperatively, suggesting that the visual cognitive ability of DNR patients was significantly deteriorated from baseline. The above findings indicated that the disorder of functional connections between the DMN and primary visual cortex might be related to visual-related cognitive impairments in patients with DNR.

In the present study, the initial RF model based on a combination of all imaging data and demographic data could satisfactorily predict DNR. Based on feature screening, the simplified RF model based on only DMN and CEN data has a marked effect on DNR classification. Compared to the initial RF model, the simplified model has the advantages of cost-efficiency and easier implementation, which are critical for the clinical prediction model. In summary, the neuroimaging data-driven machine learning recognition technique has been used for the first time in the current study to identify the patients at high risk for developing DNR, which would be beneficial for the early prevention of DNR and improving the prognosis of elderly patients post-surgery. Recently, it has been reported that the incidence of postoperative cognitive disorders in high-risk patients may be reduced by comprehensive multidisciplinary interventions, including cognitive stimulation (Álvarez et al., 2017; Deemer et al., 2020), repeated re-orientation (Colombo et al., 2012), early mobilization (Krenk et al., 2014), preoperative melatonin supplementation (Fan et al., 2017), intraoperative administration of dexmedetomidine (Cheng et al., 2019; Yang et al., 2019; Lei et al., 2020), postoperative patient-controlled epidural analgesia (Kristek et al., 2019), removal of physical restraints and catheters as permitted (Migirov et al., 2021), and family training (Mitchell et al., 2017).

Nevertheless, the present study has several limitations. First, the majority of the patients refused to undergo another rs-fMRI scan postoperatively, and hence, we were unable to find any potential aberrant FC patterns in brain networks after surgery. Second, cross-validation within the same dataset might lead to inaccurate estimation of the prediction error (Varoquaux et al., 2017; Zhu et al., 2020), rendering it uncertain whether the rs-fMRI biomarkers in this study could be extended to a broad population. Therefore, in the future study, we would collect new independent testing data (multi-center) to verify the stability and reliability of the model. Thirdly, the present study used seed-based FC analysis to explore the connectivity patterns between the core seed points of the DMN, SN, CEN or limbic network and all voxels of the whole brain. The whole-brain voxel-wise FC of other brain regions was not investigated, and it is possible to improve the performance of the prediction model by including more brain regions of other networks. Fourthly, this study is a reanalysis of our previously published data (Jiang et al., 2020). A previous study found that the DNR patients exhibited altered spontaneous neural activity in the bilateral MCC prior to surgery. rs-fMRI data could be utilized to extract maximal information. Therefore, the present study further used FC analysis to explore the preoperative connectivity patterns of cognitive-related brain networks in patients who developed DNR following non-cardiac surgery. Thus, a large group of subjects is required to substantiate the current findings in the future independent study.

## Data Availability Statement

The original contributions presented in the study are included in the article/Supplementary Material, further inquiries can be directed to the corresponding authors.

## Ethics Statement

The studies involving human participants were reviewed and approved by Huadong Hospital Affiliated to Fudan University. The patients/participants provided their written informed consent to participate in this study.

## Author Contributions

ZJ and YC made substantial contributions to the design, acquisition, analysis and interpretation of study data, and drafted the manuscript. XZ made substantial contributions to the design, acquisition and analysis of study data, and provided critical revisions to the manuscript. YL made substantial contributions to the design, analysis and interpretation of study data, and provided critical revisions to the manuscript. MZ and SLi made substantial contributions to the acquisition of study data and provided critical revisions to the manuscript. GL and ZB made substantial contributions to the interpretation of study data and provided critical revisions to the manuscript. SLiu and WG made substantial contributions to the conception, design, acquisition, analysis and interpretation of study data, drafted the manuscript, and provided critical revisions to the manuscript. All authors agreed to be accountable for all aspects of the work and approved the final manuscript.

## Conflict of Interest

The authors declare that the research was conducted in the absence of any commercial or financial relationships that could be construed as a potential conflict of interest.

## Publisher’s Note

All claims expressed in this article are solely those of the authors and do not necessarily represent those of their affiliated organizations, or those of the publisher, the editors and the reviewers. Any product that may be evaluated in this article, or claim that may be made by its manufacturer, is not guaranteed or endorsed by the publisher.

## Footnotes

1
http://www.chictr.org.cn
