# Supplementary material for: Predicting Delayed Neurocognitive Recovery After Non-cardiac Surgery Using Resting-State Brain Network Patterns Combined With Machine Learning
Source: Front Aging Neurosci. 2021 Nov 12;13:715517. doi: 10.3389/fnagi.2021.715517 (PMC8633536; doi:10.3389/fnagi.2021.715517)
Supplement: Supplementary file 1 [file Data_Sheet_1.docx]

**Supplemental Table 1. The MNI coordinates of ROIs in the DMN, SN, CEN, and limbic network**

| **ROIs** | **MNI coordinates**  **(x/y/z mm)** | | | **ROIs** | **MNI coordinates**  **(x/y/z mm)** | | |
| --- | --- | --- | --- | --- | --- | --- | --- |
| **Default mode network (DMN)** | | | | **Central executive network (CEN)** | | | |
| posterior cingulate cortex.R | 7 | -42 | 22 | anterior  prefrontal cortex.R | 44 | 45 | 0 |
| posterior cingulate cortex.L | -7 | -43 | 25 | anterior  prefrontal cortex.L | -44 | 45 | 0 |
| media prefrontal cortex.R | 2 | 61 | 13 | superior parietal lobule.R | 50 | -51 | 45 |
| media prefrontal cortex.L | -2 | 43 | -11 | superior parietal lobule.L | -50 | -51 | 45 |
| lateral parietal cortex.R | 49 | -63 | 33 | **Limbic network** | | | |
| lateral parietal cortex.L | -46 | -66 | 30 | ventral hippocampus.R | 27 | -15 | -18 |
| **Salience network (SN)** | | | | ventral hippocampus.L | -27 | -15 | -18 |
| dorsal anterior cingulate cortex.R | 6 | 21 | 36 | amygdala.R | 19 | -2 | -21 |
| dorsal anterior cingulate cortex.L | -6 | 21 | 36 | amygdala.L | -19 | -2 | -21 |
| insula.R | 41 | 3 | 6 |  |  |  |  |
| insula.L | -41 | 3 | 6 |  |  |  |  |

Abbreviations: CEN, central executive network; DMN, default mode network; MNI, Montreal Neurological Institute; ROIs, regions of interest; SN, salience network; R, right; L, left.

**
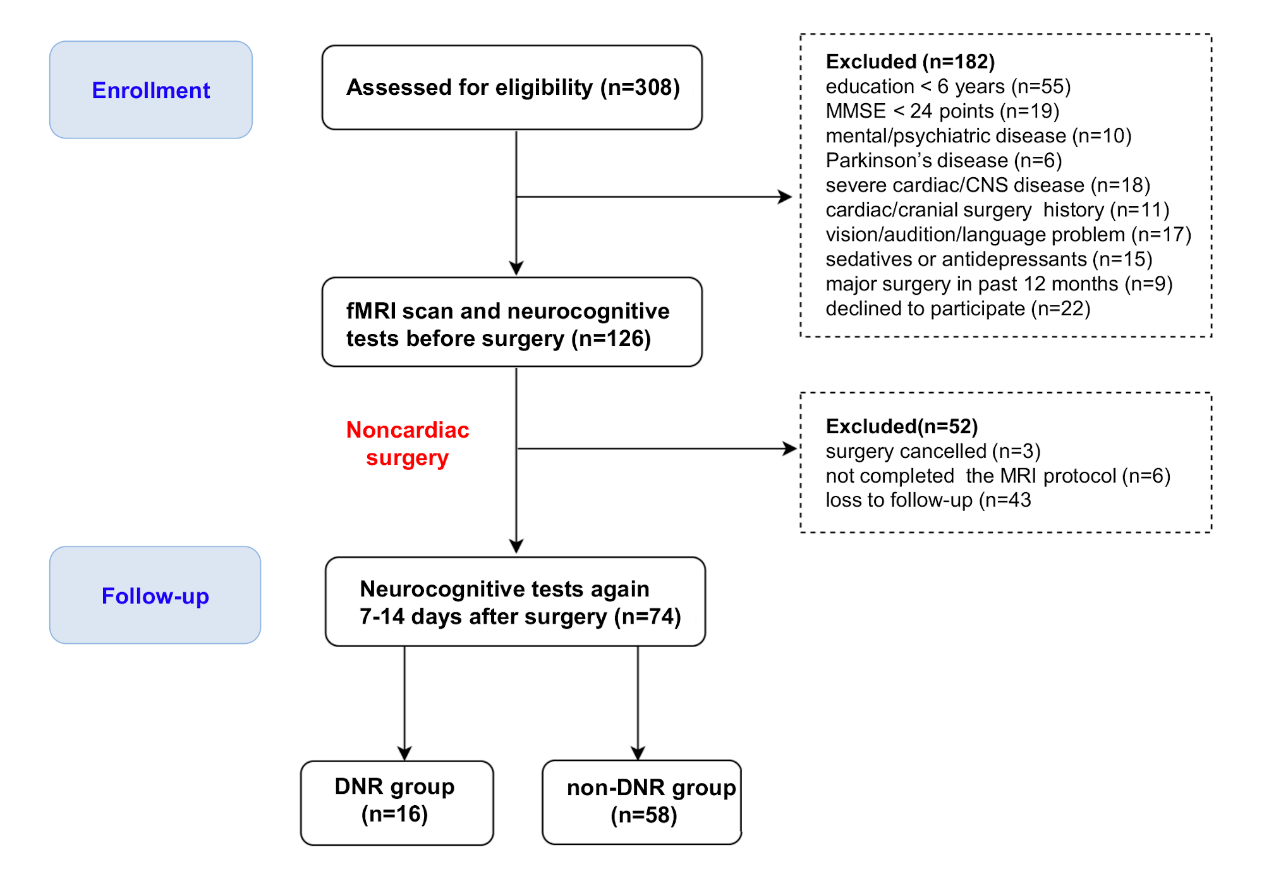
**

**Supplemental Figure 1.** Study design and flow chart.

Abbreviations: CNS, central nervous system; DNR, delayed neurocognitive recovery; fMRI: functional magnetic resonance imaging; MMSE: mini-mental state examination.
